# The Mechanism of Antimicrobial Activity of Conjugated Bile Acids against Lactic Acid Bacilli

**DOI:** 10.3390/microorganisms11071823

**Published:** 2023-07-17

**Authors:** Li-Na Chai, Hua Wu, Xue-Jiao Wang, Li-Juan He, Chun-Feng Guo

**Affiliations:** College of Food Science and Engineering, Northwest A&F University, Yangling, Xianyang 712100, China

**Keywords:** conjugated bile acid, antimicrobial activity, membrane integrity, transmembrane potential, intracellular ATP

## Abstract

The mechanism underlying antimicrobial activity of conjugated bile acids against strains of lactic acid bacilli is not well understood. The purpose of this study was to investigate two typical conjugated bile acids (glycochenodeoxycholic acid and taurochenodeoxycholic acid) for their mechanisms of antimicrobial activity against four strains of different species of lactic acid bacilli at the physiological pH of the small intestine of humans. The bacterial cell membrane integrity, transmembrane potential, and transmembrane pH gradient were examined using the fluorescence probes SYTO 9 plus propidium iodide, 3,3′-dipropylthiadicarbocyanine iodide, and 5(6)-carboxyfluorescein diacetate N-succinimidyl ester, respectively. The intracellular ATP levels were measured by the firefly luciferase-based bioluminescence method. It was found that the antimicrobial activity of conjugated bile acids against the strains of lactic acid bacilli is strain-specific, and glycochenodeoxycholic acid showed significantly greater antimicrobial activity than taurochenodeoxycholic acid against the strains of lactic acid bacilli. The conjugated bile acids inhibited the growth of strains of lactic acid bacilli by disrupting membrane integrity, dissipating transmembrane potential, reducing the transmembrane pH gradient, and depleting intracellular ATP. In conclusion, the antimicrobial activity of conjugated bile acids against lactic acid bacilli is a multifactorial phenomenon. This study will provide valuable information for developing strategies to improve the ability of lactic acid bacilli to tolerate bile in vivo.

## 1. Introduction

Probiotics are live microorganisms that confer health benefits on the host when administered in adequate amounts. Lactic acid bacteria are one of the most commonly used probiotic microorganisms [1]. Randomized controlled trials have revealed the various health benefits of the consumption of lactic acid bacilli, including the enhancement of body immunity [2], prevention and treatment of inflammatory bowel disease [3], alleviation of irritable bowel syndrome [4], regulation of intestinal flora homeostasis [5], modulation of lipid metabolisms [6], as well as prevention of colorectal cancer [7].

Lactic acid bacilli as probiotic microorganisms are typically characterized by their superior ability to survive in the gastrointestinal tract. Therefore, bile tolerance is often considered an important selection criterion for potential probiotic strains of lactic acid bacilli because bile acids in the small intestine have a detergent-like property and inhibit the growth of lactic acid bacilli [8]. Besides serving as digestive aids and microbial growth inhibitors, bile acids also play a key role in the regulation of metabolism and immune response of the body [9]. Bile acids play an important role in maintaining the balance of intestinal flora by stimulating the growth of bacteria with enzymes involved in bile acid metabolism (such as bile salt hydrolases) and inhibiting the growth of those that are sensitive to bile acids [10,11].

Bile acids are synthesized in the liver and constitute approximately 50% of the organic components of biliary bile [12]. Following a meal, the concentration of bile acids increases to 12 mM in the duodenum and then decreases to 2 mM in the ileum due to active reabsorption of bile acids [13,14]. The biliary bile acids are mainly presented in the form of the glycine and taurine conjugates of cholic, deoxycholic, and chenodeoxycholic acids, including glycocholic acid (GCA), taurocholic acid (TCA), glycodeoxycholic acid (GDCA), taurodeoxycholic acid (TDCA), glycochenodeoxycholic acid (GCDCA), and taurochenodeoxycholic acid (TCDCA), with an approximate molar ration of 24:12:16:8:24:12 [15]. GCDCA has both the highest relative content and the greatest antimicrobial activity among the three glycine-conjugated bile acids, while TCDCA has both the highest relative content and the greatest antimicrobial activity among the three taurine-conjugated bile acids [15,16].

The tolerance of lactic acid bacilli to bile acids is strain-specific [17]. Various bile resistance mechanisms in strains of lactic acid bacilli have been reported in the literature [18,19,20]. Bile acids may exert their antimicrobial activity by inducing stress response, disrupting cell membrane integrity, and destroying the transmembrane proton motive force (PMF) of microbial cells [21,22,23,24]. However, the majority of studies on antimicrobial mechanisms of bile acids focus on free bile acids. The mechanisms by which conjugated bile acids inhibit the growth of lactic acid bacilli have not been clarified, although this phenomenon was reported for a long time. In fact, this knowledge is critical for the development of strategies for the targeted intervention of the capacity of strains of lactic acid bacilli to tolerate conjugated bile acids in the small intestine, where conjugated bile acids are present at higher concentrations [25]. This may overcome the disadvantages of the traditional methods for screening for bile-tolerant strains of lactic acid bacilli, which are characterized by low efficiency and high labor intensity.

The purpose of this study was to investigate the mechanism responsible for the growth inhibition of lactic acid bacilli by two typical conjugated bile acids, GCDCA and TCDCA. The results from this study will provide helpful ideas for developing strategies to directionally regulate and control the bile tolerance of lactic acid bacilli in the human intestine.

## 2. Materials and Methods

### 2.1. Source and Maintenance of Bacterial Strains

The four strains of lactic acid bacilli used in this study were obtained from the American Type Culture Collection (ATCC, Manassas, VA, USA). These strains include *Lactiplantibacillus plantarum* ATCC 14917, *Lacticaseibacillus casei* ATCC 334, *Lacticaseibacillus rhamnosus* ATCC 53103 (GG), and *Lactobacillus acidophilus* ATCC 700396 (NCFM). Cultures of lactic acid bacilli were routinely stored at −80 °C in MRS broth (Oxoid, Basingstoke, Hampshire, UK) containing 30% (vol/vol) glycerol. The strains were maintained by subculturing with 1% (vol/vol) inocula in MRS broth supplemented with 0.5 g/L L-cysteine hydrochloride (Sigma-Aldrich, St. Louis, MO, USA) and then incubating for 16 h at 37 °C. The cultures were subcultured three times in a similar manner prior to use.

### 2.2. Antimicrobial Activity of Conjugated Bile Acids 

The conjugated bile acids were evaluated for their antimicrobial activity against strains of lactic acid bacilli using our previous method [17] with minor modifications. Briefly, an overnight culture was inoculated (0.1‱, vol/vol) into sterile MRS broth buffered with 0.1 M HEPES at a final pH 7.30 supplemented with or without a 5 mM conjugated bile acid (GCDCA or TCDCA) and incubated at 37 °C for 12 h under anaerobic conditions. The total viable count was determined using the pour plate method on MRS agar with incubation for 72 h at 37 °C under anaerobic conditions. The bile-antimicrobial index (BAI, %) was calculated using the following formula to evaluate the antimicrobial activity of conjugated bile acids.

$$BAI\left(\%\right)=\frac{\log_{2}\frac{N_{t}}{N_{0}}}{\log_{2}\frac{N_{c}}{N_{0}}}\times 100$$

where *N*_0_ represents the viable counts in the broth at zero time, and *N*_t_ and *N*_c_ represent the viable counts in the broth with and without the bile acids after anaerobic incubation, respectively.

### 2.3. Effect of Conjugated Bile Acids on Cellular Membrane Integrity

#### 2.3.1. Culturing Conditions of Strains 

The bacterial cells were harvested by centrifugation at 12,000× *g* for 10 min, and the cell pellets were washed twice with physiological saline and resuspended in a 50 mM HEPES buffer (pH 7.3) containing 20 mM glucose to an optical dispersion at 600 nm (OD_600 nm_) of 0.3. The suspensions were mixed with the same volume of GCDCA or TCDCA (Sigma-Aldrich) solution with a final bile acid concentration of 5 and 10 mM, respectively. Ultrapure water instead of the bile acid solution was used as the control. Cells were harvested after incubation at 37 °C for 1 h, washed twice with physiological saline, and resuspended in the same volume of physiological saline. 

#### 2.3.2. Determination of Cellular Membrane Integrity

The bacterial cellular membrane integrity was determined by fluorimetry, as described previously [26] with minor modifications. Briefly, the suspensions (100 μL) were mixed with the same volume of a fluorescent dye mixture (10 μM green-fluorescent nucleic acid stain SYTO 9 plus 60 μM red-fluorescent nucleic acid stain propidium iodide [PI]) of a LIVE/DEAD BacLight Bacterial Viability Kit (L7012; Thermo Fisher Scientific, Waltham, MA, USA) in a well of a black 96-well flat-bottom microtiter plate. Following 15 min of incubation at room temperature in the dark, the suspensions were excited by 485-nm-wavelength light, and the fluorescence intensity emitted at 530 and 630 nm was measured using a multimode microplate reader (Spark; Tecan, Mannedorf, Switzerland), respectively. The ratio of green to red emission spectrum intensity was calculated to represent cellular membrane integrity. To construct a calibration curve, the rate of green to red emission spectrum intensity was plotted versus the known percentage (0, 10%, 50%, 90%, and 100%) of the standard live cell suspensions, which were prepared using a 100% live cell suspension (no treatment) and a 100% dead cell suspension (treated with 70% isopropanol for 1 h, washed twice with physiological saline, and resuspended in the same volume of physiological saline) according to the manufacturer’s recommendation. 

### 2.4. Effect of Conjugated Bile Acids on Transmembrane Potential (ΔΨ)

#### 2.4.1. Culturing Conditions of Strains 

The harvested cells were washed twice with physiological saline and resuspended in a 50 mM potassium phosphate buffer (pH 7.3) supplemented with 65 U/mL catalase, 1 mM MgSO_4_, and 10 mM glucose to an OD_600 nm_ of 0.05. The suspensions (160 μL) were mixed with 20 μL of 5 μM membrane potential-sensitive fluorescent probe 3,3’-dipropylthiadicarbocyanine iodide (DiSC3(5); Aladdin, Shanghai, China) dissolved in 10% dimethyl sulfoxide (DMSO) in the wells of a black 96-well flat-bottom microtiter plate [27]. Subsequently, 20 μL of GCDCA or TCDCA solution were pipetted into the wells with a final bile acid concentration of 2.5, 5, and 10 mM, respectively. The cultures were incubated for 1 h at 37 °C in the dark. Ultrapure water instead of the bile acid solution was used as the control. 

#### 2.4.2. Determination of ΔΨ

The suspensions after incubation were used for fluorescence intensity measurement. The fluorescence intensity was measured using a Tecan Spark multimode microplate reader with excitation and emission wavelengths of 622 and 670 nm, respectively [28]. There was a negative correlation between the membrane potential and the fluorescence intensity emitted. In general, DiSC3(5) is taken up by bacteria and accumulates on hyperpolarized membranes, resulting in weak fluorescence intensity. As soon as the membrane is depolarized, the dye is released into solution with a rapidly increasing fluorescence intensity [29]. An increase in the fluorescence intensity implies that the membrane potential has dissipated [30]. A higher fluorescence intensity indicates a higher degree of membrane potential dissipation.

### 2.5. Effect of Conjugated Bile Acids on Transmembrane pH Gradient (ΔpH)

#### 2.5.1. Culturing Conditions of Strains 

The bacterial cells were harvested by centrifugation at 12,000× *g* for 10 min, and the pellets were washed twice with a 50 mM potassium phosphate buffer (pH 7.3) supplemented with 1 mM MgSO_4_ and 0.1 U/mL horseradish peroxidase (buffer A) and resuspended in buffer A supplemented with 10 mM glucose and 4 μM 5(6)-carboxyfluorescein diacetate N-succinimidyl ester (pre-dissolved in DMSO) to an OD_600 nm_ of 0.5 [31]. The suspensions were incubated in the dark at 37 °C for 30 min for fluorescent labeling. Subsequently, the cells were harvested by centrifugation at 12,000× *g* for 10 min, resuspended in the same volume of 10 mM glucose solution, and incubated for 1 h to facilitate the efflux of intracellular unconjugated probes. The cells were harvested by centrifugation again and resuspended in the same volume of buffer A supplemented with 10 mM glucose. The suspensions (180 μL) were pipetted into the wells of a black 96-well flat-bottom microtiter plate, and 20 μL of GCDCA or TCDCA solution were added to the wells with final bile acid concentrations of 2.5, 5, and 10 mM, respectively. The cultures were incubated for 30 min at 37 °C. Ultrapure water instead of the bile acid solution was used as the control. 

#### 2.5.2. Determination of Intracellular pH (pH_in_) and ΔpH

The cultures were excited using 440- and 490-nm-wavelength light, respectively, and the fluorescence emission intensity was recorded at 525 nm using a Tecan Spark multimode microplate reader [32]. The ratios of the fluorescence emission intensity from bacterial cells stained with the probes excited at 490 and 440 nm were calculated. To establish the relationship between the fluorescence emission intensity ratio and pH_in_, a calibration curve was constructed using buffer A that was adjusted to pH 6.0, 6.5, 7.0, and 7.5, respectively. The intracellular and extracellular pH values were equalized by the addition of 10 μL valinomycin (200 μM, dissolved in 10% DMSO) plus 10 μL nigericin (200 μM, dissolved in 10% DMSO). The ΔpH was calculated by subtracting extracellular pH (pH_ex_, 7.3) from the pH_in_.

### 2.6. Effect of Conjugated Bile Acids on Intracellular ATP Levels

#### 2.6.1. Culturing Conditions of Strains 

The bacterial cells were harvested by centrifugation at 12,000× *g* for 10 min, and the pellets were washed twice with a 50 mM potassium phosphate buffer (pH 7.3) supplemented with 10 mM glucose and 1 mM MgSO_4_ and resuspended in the above buffer to an OD_600 nm_ of 0.6. The suspensions (450 μL) were mixed with 50 μL of GCDCA or TCDCA solution with final bile acid concentrations of 2.5, 5 and 10 mM, respectively. The cultures were incubated for 1 h at 37 °C. Ultrapure water instead of the bile acid solution was used as the control.

#### 2.6.2. Determination of Intracellular ATP 

The intracellular ATP levels were measured using a firefly luciferase-based bioluminescence ATP Assay Kit (Beyotime Biotechnology, Shanghai, China) as described previously [33]. Briefly, the cultures were centrifuged at 12,000× *g* for 10 min at 4 °C, and the pellets were lysed with 0.5 mL of cell lysis buffer according to the manufacturer’s instructions. The lysates were centrifugated at 12,000× *g* for 5 min at 4 °C. The supernatants containing ATP were collected and stored at 4 °C until measurement. The measurement was carried out on a black 96-well flat-bottom microtiter plate. The ATP detection reagent and ATP detection diluent were dissolved on ice to prepare the ATP assay mix. The ATP assay mix (100 μL) was pipetted into the wells and kept for 10 min to reduce background fluorescence, and the lysate aliquots (20 μL) were added to each well. Following shaking for 10 s, background light emission was measured using a Tecan Spark multimode microplate reader in luminescence mode.

### 2.7. Statistical Analysis

All experiments were repeated three times in duplication, and results were expressed as mean ± standard deviation (SD). All statistical analyses except correlation analysis were performed using the SPSS statistical software package version 25.0 (IBM, Armonk, NY, USA). Differences between means were analyzed by one-way analysis of variance (ANOVA), followed by Tukey’s multiple comparison tests. A *p*-value < 0.05 was considered statistically significant. The correlation between the variables mentioned above was calculated by the Pearson correlation coefficient (r) and visualized by heatmap using GraphPad Prism software version 9.0 (GraphPad Software, San Diego, CA, USA).

## 3. Results

### 3.1. Antimicrobial Activity of Conjugated Bile Acids 

**Figure 1:**
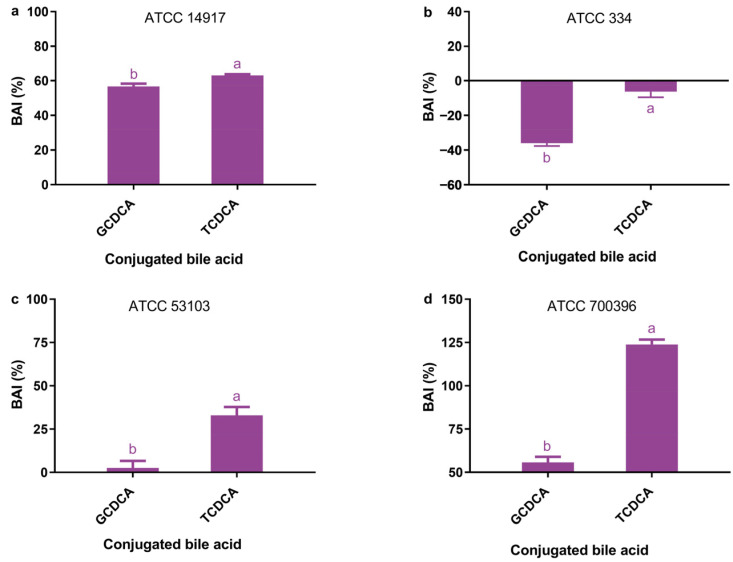
Antimicrobial activity of GCDCA and TCDCA against *Lactiplantibacillus plantarum* ATCC 14917 (**a**), *Lacticaseibacillus casei* ATCC 334 (**b**), *Lacticaseibacillus rhamnosus* ATCC 53103 (**c**), and *Lactobacillus acidophilus* ATCC 700396 (**d**). Each bile acid was used at a concentration of 5 mM. Data are expressed as the mean ± SD (*n* = 3). Means not sharing a common letter differ significantly (*p* < 0.05).

GCDCA and TCDCA showed significant differences in their antimicrobial activity (*p* < 0.05; Figure 1). In each strain of lactic acid bacilli, GCDCA showed significantly greater antimicrobial activity than TCDCA (*p* < 0.05). 

Neither GCDCA nor TCDCA exhibited bactericidal activity against strains ATCC 14917, ATCC 53103, and ATCC 700396, and they only inhibited the growth of these strains to a limited extent (Figure 1a,c,d). However, both GCDCA and TCDCA revealed evident bactericidal activity against strain ATCC 334 (Figure 1b). Overall, strain ATCC 334 was more susceptible to bile acid exposure than the other strains (Figure 1b), whereas strains ATCC 14917 and ATCC 700396 were less susceptible (Figure 1a,d).

### 3.2. Effect of Conjugated Bile Acids on Cellular Membrane Integrity 

**Figure 2:**
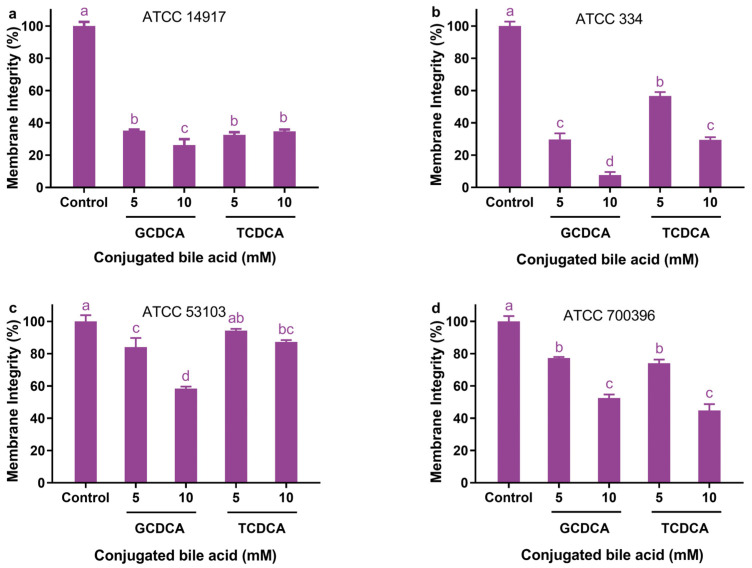
Effects of different concentrations of GCDCA and TCDCA on the cellular membrane integrity of *Lactiplantibacillus plantarum* ATCC 14917 (**a**), *Lacticaseibacillus casei* ATCC 334 (**b**), *Lacticaseibacillus rhamnosus* ATCC 53103 (**c**), and *Lactobacillus acidophilus* ATCC 700396 (**d**). Data are expressed as the mean ± SD (*n* = 3). Means not sharing a common letter differ significantly (*p* < 0.05).

Both GCDCA and TCDCA were able to disrupt cellular membrane integrity, with disruption rates varying between 5% and 92% depending on the types and concentrations of bile acids used and the strains tested (Figure 2). Generally, GCDCA appears to disrupt cellular membrane integrity more strongly than TCDCA, particularly in the presence of 10 mM of bile acids. When a bile acid concentration of 5 mM was used, this phenomenon was observed only in strains ATCC 334 and ATCC 53103 (Figure 2b,c), but not in strains ATCC 14917 and ATCC 700396 (Figure 2a,d). However, when a bile acid concentration of 10 mM was used, this phenomenon was found in three of the four strains, including ATCC 14917, ATCC 334, and ATCC 53103.

In all the strains, GCDCA showed a significantly greater ability to reduce membrane integrity at 10 mM than at 5 mM. However, the ability did not double with a 2-fold GCDCA concentration. It was also found that TCDCA reduced membrane integrity more effectively at 10 mM than at 5 mM in strains ATCC 334 and ATCC 700396 (Figure 2b,d), but not in strains ATCC 14917 and ATCC 53103 (Figure 2a,c). The ability of TCDCA to disrupt the membrane integrity of strains ATCC 14917 and ATCC 53103 did not differ significantly between 5 and 10 mM.

In addition, conjugated bile acids have a strain-specific effect on the integrity of cell membranes. As a result of bile acid exposure, the integrity of the cell membrane of strains ATCC 14917 and ATCC 334 was highly sensitive to bile acid (Figure 2a,b), whereas strains ATCC 53103 and ATCC 700396 were relatively insensitive (Figure 2c,d).

### 3.3. Effect of Conjugated Bile Acids on Cellular ΔΨ 

**Figure 3:**
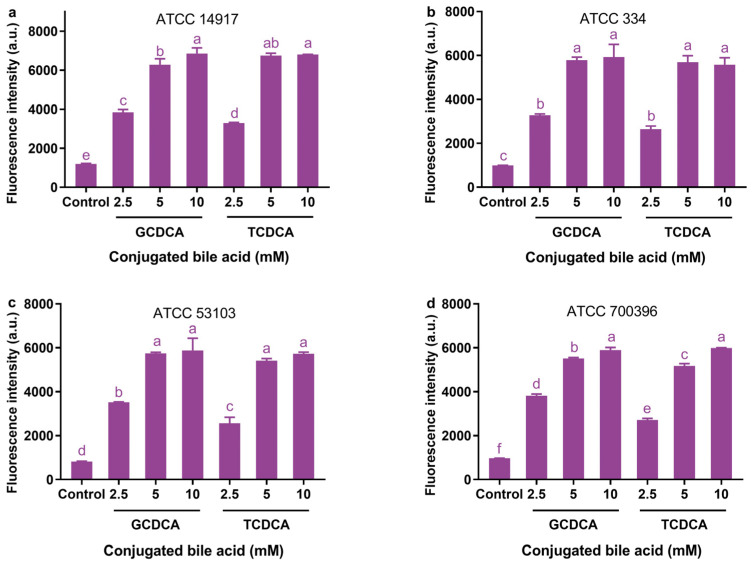
Effects of different concentrations of GCDCA and TCDCA on the ΔΨ of *Lactiplantibacillus plantarum* ATCC 14917 (**a**), *Lacticaseibacillus casei* ATCC 334 (**b**), *Lacticaseibacillus rhamnosus* ATCC 53103 (**c**), and *Lactobacillus acidophilus* ATCC 700396 (**d**). Data are expressed as the mean ± SD (*n* = 3). Means not sharing a common letter differ significantly (*p* < 0.05).

Both GCDCA and TCDCA significantly increased the fluorescence intensity of bacterial cells in all the strains, even at the lowest bile acid concentration of 2.5 mM (Figure 3). This indicates that both bile acid conjugates have greater potential to promote the dissipation of ΔΨ the bacterial cells. When exposed to 2.5 mM GCDCA, ATCC 14917, ATCC 334, ATCC 53103, and ATCC 700396 cells showed 3.2-, 3.3-, 4.2-, and 4.0-fold greater fluorescence intensity than controls, respectively. However, when exposed to 5 mM GCDCA, these bacterial cells exhibited 5.2-, 5.8-, 6.9-, and 5.7-fold greater fluorescence intensity than controls, respectively. When GCDCA concentrations were increased from 5 to 10 mM, ATCC 334 and ATCC 53103 cells did not show a significant increase in fluorescence intensity (*p* > 0.05, Figure 3b,c), but ATCC 14917 and ATCC 700396 cells exhibited a significant increase in fluorescence intensity of 9.0% and 6.9%, respectively (*p <* 0.05, Figure 3a,d).

TCDCA was found to have a very similar effect on the fluorescence intensity of bacterial cells compared with GCDCA. The fluorescence intensity of bacterial cells of each strain was not significantly different between TCDCA and GCDCA at a concentration of 10 mM (*p* > 0.05). The fluorescence intensity of only ATCC 700396 cells differed significantly between the TCDCA and GCDCA treatments at 5 mM (*p* > 0.05). Nevertheless, the GCDCA treatment only increased the fluorescence intensity of ATCC 700396 cells by 6.7% as compared with the TCDCA treatment. In contrast, the differences between the two treatments were slightly larger at 2.5 mM. As compared to the TCDCA treatment, the GCDCA treatment significantly increased the fluorescence intensity of ATCC 14917, ATCC 53103, and ATCC 700396 cells by 16.7%, 36.9%, and 20.8%, respectively (*p <* 0.05). However, there was still no significant difference in the fluorescence intensity of bacterial cells in ATCC 334 between the two treatments (*p >* 0.05).

### 3.4. Effect of Conjugated Bile Acids on ΔpH

**Figure 4:**
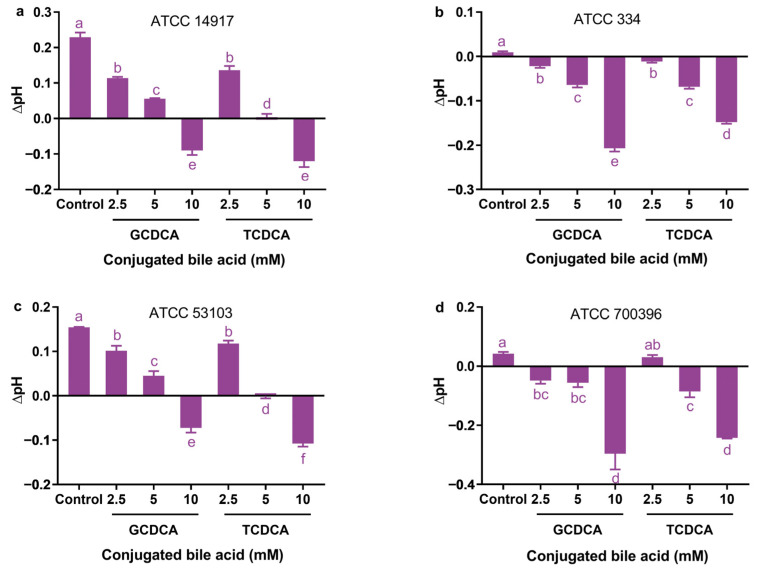
Effects of different concentrations of GCDCA and TCDCA on the cellular ΔpH of *Lactiplantibacillus plantarum* ATCC 14917 (**a**), *Lacticaseibacillus casei* ATCC 334 (**b**), *Lacticaseibacillus rhamnosus* ATCC 53103 (**c**), and *Lactobacillus acidophilus* ATCC 700396 (**d**). ΔpH = intracellular pH − extracellular pH. Data are expressed as the mean ± SD (*n* = 3). Means not sharing a common letter differ significantly (*p* < 0.05).

In the absence of GCDCA and TCDCA, strains ATCC 14917, ATCC 334, ATCC 53103, and ATCC 700396 were found to have intracellular pH (pH_in_) levels of 7.53, 7.31, 7.45, and 7.34, respectively (Figure 4a–d). However, pH_in_ levels in these strains were significantly reduced when treated with GCDCA or TCDCA at concentrations of 5 and 10 mM without exception (*p* < 0.05). In the presence of 2.5 mM GCDCA or TCDCA, all strains showed significant decreases in their pH_in_ levels compared with control cells, with the exception of strain ATCC 700396 treated with TCDCA (*p* < 0.05).

The 5 mM GCDCA treatment significantly reduced cellular ΔpH (ΔpH = pH_in_ − extracellular pH [pH_ex_]) of strains ATCC 14917, ATCC 334, ATCC 53103, and ATCC 700396 by 0.17, 0.07, 0.11, and 0.10, respectively, compared with their control cells, while the 5 mM TCDCA treatment significantly reduced cellular ΔpH of these strains by 0.23, 0.08, 0.16, and 0.13, respectively, compared with their control cells. The 10 mM GCDCA treatment significantly reduced the cellular ΔpH of these strains by 0.32, 0.22, 0.23, and 0.34, respectively, compared with their control cells, while the 10 mM TCDCA treatment significantly reduced the cellular ΔpH of these strains by 0.35, 0.16, 0.26, and 0.29, respectively, compared with their control cells.

In all the strains, the cellular ΔpH values could be flipped from a positive to a negative value as long as the concentrations of GCDCA or TCDCA reached a certain level. Strains ATCC 14917, ATCC 334, ATCC 53103, and ATCC 700396 were found to have negative ΔpH levels when the GCDCA concentration reached 10, 2.5, 10, and 2.5 mM, respectively, while these strains were found to have negative ΔpH levels when the TCDCA concentration reached 10, 2.5, 5, and 5 mM, respectively.

### 3.5. Effect of Conjugated Bile Acids on Intracellular ATP Levels 

**Figure 5:**
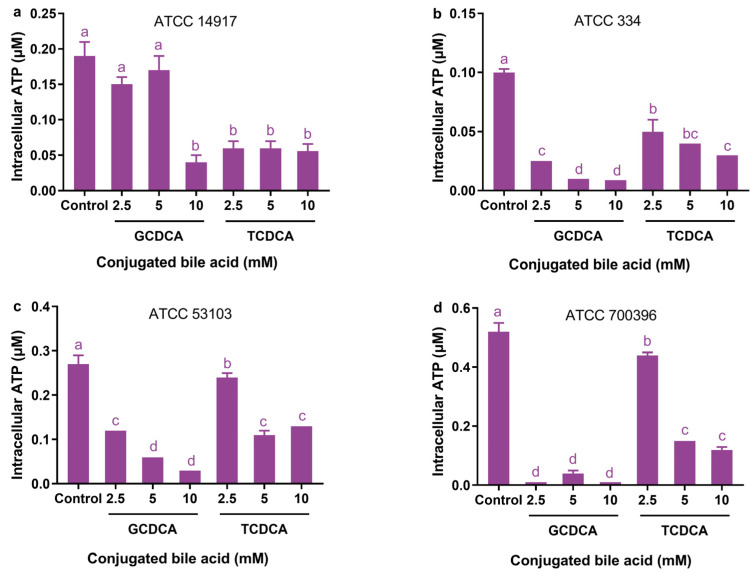
Effects of different concentrations of GCDCA and TCDCA on the intracellular ATP levels of *Lactiplantibacillus plantarum* ATCC 14917 (**a**), *Lacticaseibacillus casei* ATCC 334 (**b**), *Lacticaseibacillus rhamnosus* ATCC 53103 (**c**), and *Lactobacillus acidophilus* ATCC 700396 (**d**). Data are expressed as the mean ± SD (*n* = 3). Means not sharing a common letter differ significantly (*p* < 0.05).

Even at a concentration of 2.5 mM, both GCDCA and TCDCA treatments significantly reduced intracellular ATP levels in all strains except ATCC 14917 (*p* < 0.05; Figure 5b–d). In strain ATCC 14917, intracellular ATP levels were not significantly changed after exposure to 2.5- and 5 mM GCDCA (*p* > 0.05; Figure 5a). The 5 mM GCDCA treatment significantly reduced intracellular ATP levels of strains ATCC 334, ATCC 53103, and ATCC 700396 by 90%, 78%, and 92%, respectively, compared with their controls (*p* < 0.05), while the 10 mM GCDCA treatment significantly reduced intracellular ATP levels of these strains by 91%, 89%, and 98%, respectively, compared with their controls (*p* < 0.05). 

The TCDCA treatment was generally less effective than the GCDCA treatment in reducing intracellular ATP levels in the strains. The 5 mM TCDCA treatment significantly reduced intracellular ATP levels of strains ATCC 334, ATCC 53103, and ATCC 700396 by 60%, 59%, and 71%, respectively, compared with their controls (*p* < 0.05), while the 10 mM GCDCA treatment significantly reduced intracellular ATP levels of these strains by 70%, 52%, and 77%, respectively, compared with their controls (*p* < 0.05). 

There was considerable variation in the sensitivity of intracellular ATP levels to TCDCA and TCDCA exposure among the strains. In terms of intracellular ATP levels, strain ATCC 14917 is least sensitive to bile acid exposure, while strain ATCC 700396 is most sensitive. The 2.5 mM GCDCA treatment resulted in a 98% reduction in intracellular ATP levels in strain ATCC 700396. According to these results, neither GCDCA nor TCDCA treatments appeared to be clearly dependent on their concentrations in reducing intracellular ATP levels of the strains.

### 3.6. Correlation between the Variables

**Figure 6:**
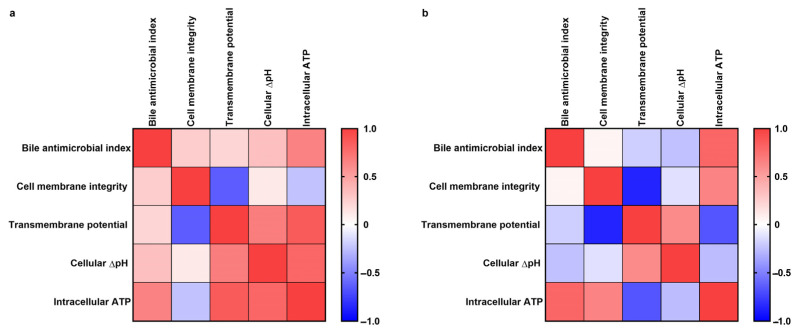
Correlation heatmap analysis between the variables. The correlation was assessed using Pearson correlation coefficients (r). Red represents a positive correlation, and blue represents a negative correlation. In subfigures (**a**) and (**b**), GCDCA and TCDCA were used at 5 mM, respectively.

Figure 6 illustrates a heatmap that depicts the correlations between any two variables: bile antimicrobial index, cellular membrane integrity, cellular transmembrane potential, cellular ΔpH, and intracellular ATP level. Unfortunately, there was no significant correlation between any two variables (*p* > 0.05). Consequently, no single pathway was able to significantly affect the growth of all the strains of lactic acid bacilli, and the antimicrobial properties of GCDCA and TCDCA should be attributed to the combination of multiple pathways.

## 4. Discussion

This study found that the exposure of conjugated bile acids caused loss of membrane integrity, dissipation of ΔΨ, dissipation or flipping of ΔpH, and depletion of the intracellular ATP pool in the strains. However, the effects of these pathways varied depending on the strains evaluated. Strains ATCC 14917 and ATCC 334 were most sensitive to conjugated bile acids with respect to their cellular membrane integrity, while strain ATCC 700396 was most sensitive to conjugated bile acids with respect to their intracellular ATP levels. It is reported that bile salt hydrolase is an intracellular enzyme in lactic acid bacteria. In this study, the intracellular pH of the strains exceeded 7.0, which is higher than the reported optimum pH for the activity of bile salt hydrolase in lactic acid bacilli [34,35,36]. As a result, the hydrolytic efficiency of bile salt hydrolase was markedly reduced. In addition, due to using lower bacterial population density and/or shorter incubation periods with GCDCA and TCDCA, the bile salt hydrolase-positive strains (ATCC 14917, ATCC 334, and ATCC 700396) did not result in marked deconjugation of GCDCA and TCDCA in the media (<5% deconjugation rate). Therefore, the effect of free bile acids can be ignored.

Bile response is a multifactorial phenomenon; there are many studies on bile acid resistance mechanisms of lactic acid bacilli strains using physiological and biochemical analysis, multi-omics analysis, and genetic engineering. Due to the existence of different bile acid resistance mechanisms, strains of lactic acid bacilli respond differently to conjugated bile acid exposure [18]. This may explain the phenomenon observed in this study: conjugated bile acids showed strain-dependent antimicrobial activity against strains of lactic acid bacilli. It has been reported that specific bile acid resistance mechanisms in lactic acid bacilli include increases in active extrusion of the bile acids that accumulate in the cytoplasm by activating bile acid efflux pumps (ATCC 700396) [37,38], hydrolysis of conjugated bile acids that enter the cytoplasm by using catalysis of intracellular bile salt hydrolases (strains ATCC 14917, ATCC 334, and ATCC 700396) [39], changes in the envelope structure by producing capsular exopolysaccharides (strain ATCC 53103) or S-layer protein (strain ATCC 700396) [40], activation of molecular machinery to counteract oxidative and acid stresses by sensing bile acid presence using two-component regulatory systems (strain ATCC 700396) [41].

Cell membranes of lactic acid bacilli are composed primarily of phospholipid bilayers, and their hydrophobic tails are located in the opposite direction of the cytoplasm and extracellular fluid [42]. Bile acids are generally believed to penetrate and accumulate in the lipid bilayer, thus exerting their antimicrobial properties [43]. It is likely that the hydrophobic property of conjugated bile acids explains the relationship between the chemical structure and the antimicrobial activity of conjugated bile acids observed in this study. Highly hydrophobic compounds are easier to diffuse into and accumulate in the lipid bilayer. The hydrophobicity indices of GCDCA and TCDCA are 0.51 and 0.46, respectively [16,44]. Thus, GCDCA is capable of penetrating more rapidly and accumulating more in the lipid bilayer than TCDCA. As a result, GCDCA has a greater ability to disrupt the cellular membrane integrity of lactic acid bacilli than TCDCA. This is a key reason why GCDCA displayed greater antimicrobial activity than TCDCA. 

Intact cell membranes are the basis for the normal physiological activities of bacteria and the main target of environmental stress [45]. GCDCA and TCDCA are similar to gallic acid and phenyllactic acid in their effects on bacterial membrane integrity. Gallic acid [32] and phenyllactic acid [46] have been reported to cause a loss of cellular membrane integrity in *Yersinia enterocolitica* and *Bacillus cereus*, respectively. 

There are numerous critical or essential functions performed by the bacterial cell membrane, which is composed of roughly equal amounts of proteins and lipids. The main membrane lipid components are phospholipids in the form of a bilayer, which constitutes the major boundary outlining the cell cytoplasm and controls the transport and diffusion of numerous small molecules and secreted proteins between the intracellular and extracellular spaces [45]. In the present study, the GCDCA and TCDCA treatments should promote incorporation of GCDCA and TCDCA into the phospholipid bilayer, which interfered with the normal arrangement of phospholipid molecules in the bilayer and thereby caused a loss of membrane integrity in the strains of lactic acid bacilli. We speculated that the GCDCA and TCDCA treatments did not cause the formation of large pores in the membrane because cellular ΔΨ and ΔpH in the four strains of lactic acid bacilli did not become zero after the treatments, even at a concentration of 10 mM. 

The transmembrane ion concentration gradient provides the required electrochemical energy for cell growth and many other cell processes (such as ATP synthesis). This energy is PMF, consisting of ΔΨ and ΔpH [47]. Changes in PMF indicate pore formation and subsequent leakage of essential ions and small molecules [48]. Unlike neutrophilic bacteria like Escherichia coli that maintain a pH_in_ close to neutral when the pH_ex_ is reduced, many lactic acid bacteria, particularly lactic acid bacilli, have developed a different strategy; the pH_in_ of these bacteria decreases as the pH_ex_ decreases during growth in order to maintain a constant ΔpH rather than a constant pH_in_ [49]. Using this strategy is advantageous to lactic acid bacteria because proton translocation consumes energy, and aerobes gain a significantly lower amount of energy from sugar metabolism than aerobes [50]. A positively applicable ΔpH is critical for the control of many cellular processes in bacteria, such as ATP synthesis, RNA and protein synthesis, DNA replication, and cell growth. It has been reported that a point occurs at which the ΔpH of lactic acid bacteria collapses or approaches zero, resulting in a loss of cell viability [51]. The decrease in cellular ΔpH caused by GCDCA and TCDCA observed in this study can be attributed to two factors: (1) loss of cell membrane integrity; (2) intracellular ionization of protonated conjugated bile acids. It has been generally accepted that organic acids can cross the bacterial cell membrane in their undissociated form and cause acidification in the cytoplasm of the living cells, leading to energy depletion and slow growth [32,52]. In a previous study, it was found that unconjugated bile acids (CA and DCA) reduced intracellular pH more than conjugated bile acids (GCA and TCA), possibly as a result of higher pKa values [23]. However, no obvious effect of the regular pattern of structure on pH_i_ was observed in this study. Despite the fact that GCDCA crosses the membrane more easily than TCDCA, it is more difficult to dissociate within the cell due to its higher pKa value [53]. This explains why GCDCA and TCDCA generally did not differ significantly in their effects on the cellular pH_in_ of strains of lactic acid bacilli. 

A wide range of bacterial physiology and behaviors are regulated by transmembrane potential, including pH homeostasis, membrane transport, motility, membrane protein conformation, antibiotic resistance, cell division, electrical communication, and environmental sensing [54]. There has been considerable evidence that many antimicrobial agents, particularly antimicrobial peptides, exert their antimicrobial activity by rapidly dissipating the bacterial ΔΨ of a wide range of spoilage and pathogenic bacteria [55,56,57]. The conjugated bile acids used in this study have similar mechanisms of action to these antimicrobial agents. They were able to significantly dissipate the ΔΨ of strains of lactic acid bacilli, even at a low concentration of 2.5 mM. The loss of membrane integrity and the decrease in pH_in_ are believed to be two important mechanisms by which GCDCA and TCDCA dissipated the ΔΨ of strains of lactic acid bacilli.

ATP, an energy-carrying molecule found in the cells of all living organisms, captures chemical energy obtained from the breakdown of nutrients (mainly carbohydrates) and releases it to fuel a broad range of cellular processes involved with cellular survival, growth, replication, and division [58]. Lactic acid bacilli produce ATP by fermenting carbohydrates in the cytoplasm, relying on substrate-level phosphorylation using homofermentation or heterofermentative pathways [59]. PMF has been demonstrated to be a direct factor determining intracellular ATP concentration in bacterial cells because it controls the active transport of molecules like carbohydrates, which are the main raw materials for the synthesis of ATP within the cell [60]. Therefore, if PMF is disrupted, the transport of carbohydrates will also be disrupted, resulting in a lower rate of ATP synthesis. Thus, PMF plays a vital role in regulating ATP production in bacterial cells. Our observation that GCDCA and TCDCA caused the decrease in pH_in_ and dissipation of ΔΨ in the strains of lactic acid bacilli means that the two conjugated bile acids resulted in the collapse of PMF in the bacterial cells because PMF = ΔΨ − ZΔpH (ΔΨ is a negative value; Z = RT/F, where R is the gas constant, T is the absolute temperature, and F is the Faraday constant) [61]. Therefore, the collapse of PMF induced by the conjugated bile acids is an important reason for the conjugated bile acids-induced decrease in intracellular ATP levels in the strains of lactic acid bacilli.

## 5. Conclusions

The antimicrobial activity of conjugated bile acids against the strains of lactic acid bacilli is strain-specific. This antimicrobial activity is a multifactorial phenomenon, and many factors were involved in this process, including loss of cellular membrane integrity, dissipation of ΔΨ, decrease in ΔpH, and depletion of intracellular ATP. The contribution of each factor to total antimicrobial activity varied among the strains. Understanding the pathways by which conjugated bile acids exert their antimicrobial activity against strains of lactic acid bacilli is helpful to develop strategies to enhance bile tolerance of strains of lactic acid bacilli in the small intestine of humans. In order to gain a deeper understanding of the molecular mechanisms involved in the above pathways, it is necessary to investigate the effects of conjugated bile acids on gene and protein expression as well as the interactions between conjugated bile acids and proteins in lactic acid bacilli.

## Data Availability

The datasets generated and/or analyzed during the current study are available from the corresponding author on reasonable request.

## References

[1] Rastogi S., Singh A. (2022). Gut microbiome and human health: Exploring how the probiotic genus *Lactobacillus* modulate immune responses. Front. Pharmacol..

[2] Roggero P., Liotto N., Pozzi C., Braga D., Troisi J., Menis C., Gianni M.L., Canani R.B., Paparo L., Nocerino R. (2020). Analysis of immune, microbiota and metabolome maturation in infants in a clinical trial of *Lactobacillus paracasei* CBA L74-fermented formula. Nat. Commun..

[3] Kazemi A., Soltani S., Ghorabi S., Keshtkar A., Daneshzad E., Nasri F., Mazloomi S.M. (2020). Effect of probiotic and synbiotic supplementation on inflammatory markers in health and disease status: A systematic review and meta-analysis of clinical trials. Clin. Nutr..

[4] Liu Y., Yu X., Yu L., Tian F., Zhao J., Zhang H., Qian L., Wang Q., Xue Z., Zhai Q. (2021). *Lactobacillus plantarum* CCFM8610 alleviates irritable bowel syndrome and prevents gut microbiota dysbiosis: A randomized, double-blind, placebo-controlled, pilot clinical trial. Engineering.

[5] Castro-Mejia J.L., O’Ferrall S., Krycha L., O’Mahony E., Namusoke H., Lanyero B., Kot W., Nabukeera-Barungi N., Michaelsen K.F., Molgaard C. (2020). Restitution of gut microbiota in Ugandan children administered with probiotics (*Lactobacillus rhamnosus* GG and *Bifidobacterium animalis* subsp. lactis BB-12) during treatment for severe acute malnutrition. Gut Microbes.

[6] Qiu X., Wu Q., Li W., Tang K., Zhang J. (2022). Effects of Lactobacillus supplementation on glycemic and lipid indices in overweight or obese adults: A systematic review and meta-analysis. Clin. Nutr..

[7] Oh N.S., Lee J.Y., Kim Y.-T., Kim S.H., Lee J.-H. (2020). Cancer-protective effect of a synbiotic combination between *Lactobacillus gasseri* 505 and a *Cudrania tricuspidataleaf* extract on colitis-associated colorectal cancer. Gut Microbes.

[8] Ayyash M.M., Abdalla A.K., AlKalbani N.S., Baig M.A., Turner M.S., Liu S.-Q., Shah N.P. (2021). Invited review: Characterization of new probiotics from dairy and nondairy products-Insights into acid tolerance, bile metabolism and tolerance, and adhesion capability. J. Dairy. Sci..

[9] Evangelakos I., Heeren J., Verkade E., Kuipers F. (2021). Role of bile acids in inflammatory liver diseases. Semin. Immunopathol..

[10] Guo X.H., Okpara E.S., Hu W.T., Yan C.Y., Wang Y., Liang Q.L., Chiang J.Y.L., Han S.X. (2022). Interactive Relationships between Intestinal Flora and Bile Acids. Int. J. Mol. Sci..

[11] Duboc H., Rainteau D., Rajca S., Humbert L., Farabos D., Maubert M., Grondin V., Jouet P., Bouhassira D., Seksik P. (2012). Increase in fecal primary bile acids and dysbiosis in patients with diarrhea-predominant irritable bowel syndrome. Neurogastroenterol. Motil..

[12] Winston J.A., Theriot C.M. (2020). Diversification of host bile acids by members of the gut microbiota. Gut Microbes.

[13] Kalantzi L., Goumas K., Kalioras V., Abrahamsson B., Dressman J.B., Reppas C. (2006). Characterization of the human upper gastrointestinal contents under conditions simulating bioavailability/bioequivalence studies. Pharm. Res..

[14] Northfield T.C., McColl I. (1973). Postprandial concentrations of free and conjugated bile acids down the length of the normal human small intestine. Gut.

[15] Staggers J.E., Hernell O., Stafford R.J., Carey M.C. (1990). Physical-chemical behavior of dietary and biliary lipids during intestinal digestion and absorption. 1. Phase behavior and aggregation states of model lipid systems patterned after aqueous duodenal contents of healthy adult human beings. Biochemistry.

[16] Wang X.-J., Chen B.-Y., Yang B.-W., Yue T.-L., Guo C.-F. (2021). Chemical structure, concentration, and pH are key factors influencing antimicrobial activity of conjugated bile acids against lactobacilli. J. Dairy. Sci..

[17] Hu P.-L., Yuan Y.-H., Yue T.-L., Guo C.-F. (2018). A new method for the *in vitro* determination of the bile tolerance of potentially probiotic lactobacilli. Appl. Microbiol. Biotechnol..

[18] Bustos A.Y., de Valdez G.F., Fadda S., Taranto M.P. (2018). New insights into bacterial bile resistance mechanisms: The role of bile salt hydrolase and its impact on human health. Food Res. Int..

[19] Ruiz L., Margolles A., Sanchez B. (2013). Bile resistance mechanisms in *Lactobacillus* and *Bifidobacterium*. Front. Microbiol..

[20] Bustos A.Y., de Valdez G.F., Raya R., de Almeida A.M., Fadda S., Taranto M.P. (2015). Proteomic analysis of the probiotic *Lactobacillus reuteri* CRL1098 reveals novel tolerance biomarkers to bile acid-induced stress. Food Res. Int..

[21] Bernstein H., Payne C.M., Bernstein C., Schneider J., Beard S.E., Crowley C.L. (1999). Activation of the promoters of genes associated with DNA damage, oxidative stress, ER stress and protein malfolding by the bile salt, deoxycholate. Toxicol. Lett..

[22] Kurdi P., Kawanishi K., Mizutani K., Yokota A. (2006). Mechanism of growth inhibition by free bile acids in lactobacilli and bifidobacteria. J. Bacteriol..

[23] Sannasiddappa T.H., Lund P.A., Clarke S.R. (2017). *In vitro* antibacterial activity of unconjugated and conjugated bile salts on *Staphylococcus aureus*. Front. Microbiol..

[24] Watanabe M., Fukiya S., Yokota A. (2017). Comprehensive evaluation of the bactericidal activities of free bile acids in the large intestine of humans and rodents. J. Lipid Res..

[25] Humbert L., Rainteau D., Tuvignon N., Wolf C., Seksik P., Laugier R., Carriere F. (2018). Postprandial bile acid levels in intestine and plasma reveal altered biliary circulation in chronic pancreatitis patients. J. Lipid Res..

[26] Campos F.M., Couto J.A., Figueiredo A.R., Toth I.V., Rangel A.O.S.S., Hogg T.A. (2009). Cell membrane damage induced by phenolic acids on wine lactic acid bacteria. Int. J. Food Microbiol..

[27] Dupont C., Chen Y., Xu Z., Roquet-Baneres F., Blaise M., Witt A.-K., Dubar F., Biot C., Guerardel Y., Maurer F.P. (2019). A piperidinol-containing molecule is active against Mycobacterium tuberculosis by inhibiting the mycolic acid flippase activity of MmpL3. J. Biol. Chem..

[28] Sun Y., Dong W., Sun L., Ma L., Shang D. (2015). Insights into the membrane interaction mechanism and antibacterial properties of chensinin-1b. Biomaterials.

[29] Wang Y., Wu P., Liu F., Chen J., Xue J., Qin Y., Chen F., Wang S., Ji L. (2022). Design, synthesis, and biological evaluation of membrane-active honokiol derivatives as potent antibacterial agents. Eur. J. Med. Chem..

[30] Weinhaupl K., Lindau C., Hessel A., Wang Y., Schuetze C., Jores T., Melchionda L., Schoenfisch B., Kalbacher H., Bersch B. (2018). Structural basis of membrane protein chaperoning through the mitochondrial intermembrane space. Cell.

[31] Breeuwer P., Drocourt J.L., Rombouts F.M., Abee T. (1996). A novel method for continuous determination of the intracellular pH in bacteria with the internally conjugated fluorescent probe 5 (and 6-)-carboxyfluorescein succinimidyl ester. Appl. Environ. Microb..

[32] Tian L., Fu J., Wu M., Liao S., Jia X., Wang J., Yang S., Liu Z., Liu Z., Xue Z. (2022). Evaluation of gallic acid on membrane damage of Yersinia enterocolitica and its application as a food preservative in pork. Int. J. Food Microbiol..

[33] Tian L., Wang X., Liu R., Zhang D., Wang X., Sun R., Guo W., Yang S., Li H., Gong G. (2021). Antibacterial mechanism of thymol against *Enterobacter sakazakii*. Food Control.

[34] Bi J., Fang F., Lu S., Du G., Chen J. (2013). New insight into the catalytic properties of bile salt hydrolase. J. Mol. Catal. B-Enzym..

[35] Kumar R., Rajkumar H., Kumar M., Varikuti S.R., Athimamula R., Shujauddin M., Ramagoni R., Kondapalli N. (2013). Molecular cloning, characterization and heterologous expression of bile salt hydrolase (Bsh) from *Lactobacillus fermentum* NCDO394. Mol. Biol. Rep..

[36] Li C., Ji Q., He T., Liu Y., Ma Y. (2021). Characterization of a recombinant bile salt hydrolase (BSH) from *Bifidobacterium bifidum* for its glycine-conjugated bile salts specificity. Biocatal. Biotransform..

[37] Ali S.A., Singh P., Tomar S.K., Mohanty A.K., Behare P. (2020). Proteomics fingerprints of systemic mechanisms of adaptation to bile in *Lactobacillus fermentum*. J. Proteom..

[38] Pfeiler E.A., Klaenhammer T.R. (2009). Role of transporter proteins in bile tolerance of *Lactobacillus acidophilus*. Appl. Environ. Microbiol..

[39] Wang G.Q., Yu H.I., Feng X., Tang H.Y., Xiong Z.Q., Xia Y.J., Ai L.Z., Song X. (2021). Specific bile salt hydrolase genes in *Lactobacillus plantarum* AR113 and relationship with bile salt resistance. LWT-Food Sci. Technol..

[40] Gaucher F., Bonnassie S., Rabah H., Marchand P., Blanc P., Jeantet R., Jan G. (2019). Review: Adaptation of beneficial *Propionibacteria*, *Lactobacilli*, and *Bifidobacteria* improves tolerance toward technological and digestive stresses. Front. Microbiol..

[41] Pfeiler E.A., Azcarate-Peril M.A., Klaenhammer T.R. (2007). Characterization of a novel bile-inducible operon encoding a two-component regulatory system in *Lactobacillus acidophilus*. J. Bacteriol..

[42] Fonseca F., Penicaud C., Elizabeth Tymczyszyn E., Gomez-Zavaglia A., Passot S. (2019). Factors influencing the membrane fluidity and the impact on production of lactic acid bacteria starters. Appl. Microbiol. Biotechnol..

[43] Di Gregorio M.C., Cautela J., Galantini L. (2021). Physiology and Physical Chemistry of Bile Acids. Int. J. Mol. Sci..

[44] Heuman D.M. (1989). Quantitative estimation of the hydrophilic-hydrophobic balance of mixed bile salt solutions. J. Lipid Res..

[45] Strahl H., Errington J. (2017). Bacterial Membranes: Structure, Domains, and Function. Annu. Rev. Microbiol..

[46] Ning Y., Fu Y., Hou L., Ma M., Wang Z., Li X., Jia Y. (2021). iTRAQ-based quantitative proteomic analysis of synergistic antibacterial mechanism of phenyllactic acid and lactic acid against *Bacillus cereus*. Food Res. Int..

[47] Trinh N.T.T., Dumas E., Le Thanh M., Degraeve P., Ben Amara C., Gharsallaoui A., Oulahal N. (2015). Effect of a Vietnamese *Cinnamomum cassia* essential oil and its major component trans-cinnamaldehyde on the cell viability, membrane integrity, membrane fluidity, and proton motive force of *Listeria innocua*. Can. J. Microbiol..

[48] Sun Z., Li P., Liu F., Bian H., Wang D., Wang X., Zou Y., Sun C., Xu W. (2017). Synergistic antibacterial mechanism of the *Lactobacillus crispatus* surface layer protein and nisin on Staphylococcus saprophyticus. Sci. Rep..

[49] Siegumfeldt H., Rechinger K.B., Jakobsen M. (2000). Dynamic changes of intracellular pH in individual lactic acid bacterium cells in response to a rapid drop in extracellular pH. Appl. Environ. Microbiol..

[50] Guan N., Liu L. (2020). Microbial response to acid stress: Mechanisms and applications. Appl. Microbiol. Biotechnol..

[51] Molina-Gutierrez A., Stippl V., Delgado A., Ganzle M.G., Vogel R.F. (2002). In situ determination of the intracellular pH of *Lactococcus lactis* and *Lactobacillus plantarum* during pressure treatment. Appl. Environ. Microbiol..

[52] Dijksterhuis J., Meijer M., van Doorn T., Houbraken J., Bruinenberg P. (2019). The preservative propionic acid differentially affects survival of conidia and germ tubes of feed spoilage fungi. Int. J. Food Microbiol..

[53] Carey M.C. (1984). Bile acids and bile salts: Ionization and solubility properties. Hepatology.

[54] Benarroch J.M., Asally M. (2020). The microbiologist’s guide to membrane potential dynamics. Trends Microbiol..

[55] Seydlova G., Sokol A., Liskova P., Konopasek I., Fiser R. (2019). Daptomycin pore formation and stoichiometry depend on membrane potential of target membrane. Antimicrob. Agents Chemother..

[56] Schilling N.A., Berscheid A., Schumacher J., Saur J.S., Konnerth M.C., Wirtz S.N., Beltran-Belena J.M., Zipperer A., Krismer B., Peschel A. (2019). Synthetic Lugdunin analogues reveal essential structural motifs for antimicrobial action and proton translocation capability. Angew. Chem-Int. Edit.

[57] Shen B.-Y., Wang M.-M., Xu S.-M., Gao C., Wang M., Li S., Ampomah-Wireko M., Chen S.-C., Yan D.-C., Qin S. (2022). Antibacterial efficacy evaluation and mechanism probe of small lysine chalcone peptide mimics. Eur. J. Med. Chem..

[58] Deng Y., Beahm D.R., Ionov S., Sarpeshkar R. (2021). Measuring and modeling energy and power consumption in living microbial cells with a synthetic ATP reporter. BMC Biol..

[59] Suissa R., Oved R., Jankelowitz G., Turjeman S., Koren O., Kolodkin-Gal I. (2022). Molecular genetics for probiotic engineering: Dissecting lactic acid bacteria. Trends Microbiol..

[60] Rao S.P.S., Alonso S., Rand L., Dick T., Pethe K. (2008). The protonmotive force is required for maintaining ATP homeostasis and viability of hypoxic, nonreplicating *Mycobacterium tuberculosis*. Proc. Natl. Acad. Sci. USA.

[61] Bruno M.E.C., Montville T.J. (1993). Common mechanistic action of bacteriocins from lactic-acid bacteria. Appl. Environ. Microbiol..

